# Treatment of granulomatous rosacea with adalimumab

**DOI:** 10.1016/j.jdcr.2023.08.017

**Published:** 2023-08-25

**Authors:** Ryan C. Saal, Luis J. Borda, Melissa L. Hoffman, Alice A. Roberts, Abby S. Van Voorhees

**Affiliations:** a School of Medicine, Eastern Virginia Medical School, Norfolk, Virginia; b Department of Dermatology, Eastern Virginia Medical School, Norfolk, Virginia

**Keywords:** adalimumab, granuloma, granulomatous rosacea, TNF-alpha inhibitor

## Introduction

Granulomatous rosacea (GR) is a chronic condition that manifests with yellow, brown, and red nodules and papules with erythema and is characterized histologically by a granulomatous infiltrate.^1^^,^^2^ Standardized treatment regimens are lacking. We report a recalcitrant case of GR that responded to adalimumab indicating a potential new treatment alternative for this condition.

## Case report

A 41-year-old woman presented with a 12-year history of discoloration on the nose. Twelve years prior, the patient reported that this lesion began as a painful, red, draining papule. Past medical treatments included isotretinoin, doxycycline, oral metronidazole, topical clindamycin, topical metronidazole, topical permethrin, tretinoin cream, hydroquinone 4% cream, and hydrocortisone 2.5% cream, all of which resulted in no improvement. The development of pustules and papules on the nose with yellow drainage and crusting continued over time. Medial history is notable for hypertension, iron deficiency anemia, and seasonal allergies. Surgical history includes gastric sleeve and C-section.

**Fig 1:**
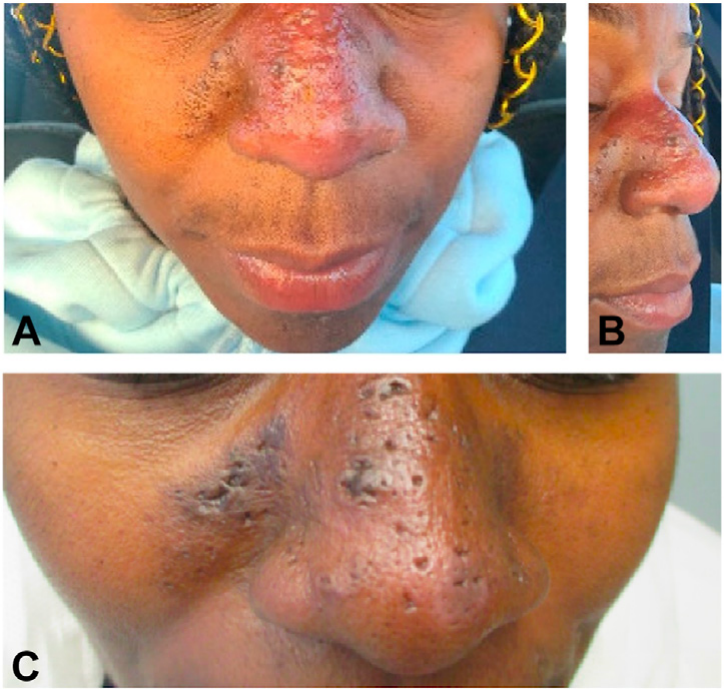
Clinical manifestations. **A-B,** Presentation before adalimumab therapy. Indurated papules and cysts coalescing into a large plaque with early rhinophymatous changes and scarring located on the nasal dorsum extending to bilateral nasolabial folds and mid-face. **C,** Presentation after adalimumab therapy. Hyperpigmented, thin plaques with atrophic scars and subtle erythema with reduced edema and tenderness of the nasal dorsum and mid-face, after 3 months of treatment.

**Fig 2:**
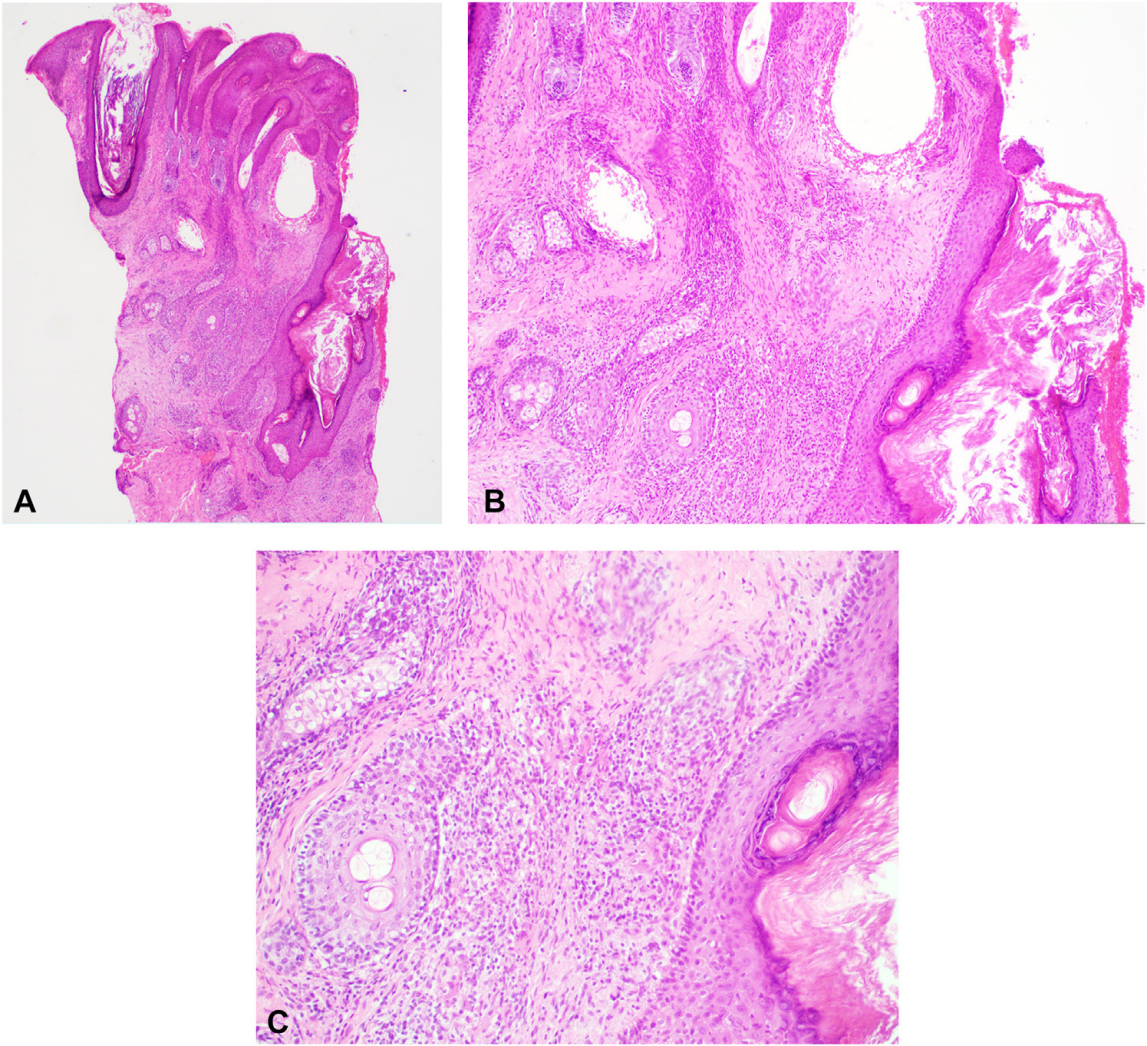
Histology findings (H&E stain). **A,** Dilation of hair follicles and telangiectatic blood vessel in right upper dermis at low power (×40). **B,** Perifollicular inflammation and vascular telangiectasia at medium power (×100). **C,** Prominent lymphohistiocytic/granulomatous inflammation near the hair follicle (×200).

Physical examination revealed indurated papules and cysts coalescing into a large plaque with a scar located on the nasal dorsum extending to bilateral nasolabial folds and mid-face. Early rhinophymatous change of the nose were also noted (Fig 1, *A*-*B*). No sinus tract involvement was seen. Punch biopsy showed a dilated follicular infundibulum with a perifollicular and perivascular lymphohistiocytic infiltrate consisting of plasma cells and a few giant cells, suggestive of granulomatous inflammation, within the dermis (Fig 2). Stains for microorganisms, including *Periodic acid–Schiff*, Acid-Fast Bacilli and Fite, were negative. Thus, a diagnosis of GR was rendered. Despite the pseudocomedone-like scars seen on the patient’s face, the lack of deep sinus tract, abscess formation, and significant numbers of plasma cells on histological examination made hidradenitis suppurativa a much less likely diagnosis.

Given the granulomatous nature of her condition, weekly, subcutaneous injections of adalimumab 40 mg were used. Three months later, physical examination showed hyperpigmented thin plaques with multifocal atrophic scars and subtle erythema of the nasal dorsum (Fig 1, *C*). Clinical improvement was noted at her 3 months follow-up appointment, given the reduction in edema, open comedo-like lesions and tenderness of her nose. No new cysts had developed since beginning this treatment. She remains on maintenance adalimumab treatment, for almost a year.

## Discussion

Rosacea can be divided up into the following subtypes according to the clinical findings: erythemato-telangiectatic, papulopustular, phymatous, and ocular.^2^^,^^3^ GR has been cited as a rosacea variant in the past, but this classification has become controversial because of its exclusion from the recent update from the National Rosacea Society.^1^^,^^2^ Regardless, GR presents with yellow, brown, and red nodules and papules with erythema located on the periorificial area and the cheeks.^2^^,^^3^ These lesions can also lead to residual scarring.^4^ On histology, a range of findings have been reported, including noncaseating granulomas, mixed lymphohistiocytic inflammation with perivascular and perifollicular infiltration, and caseating epithelioid granulomas.^3^^,^^5^

Treatments often used for GR include oral antibiotics, metronidazole, corticosteroids, benzoyl peroxide, alpha-adrenergic vasoconstrictors, dapsone, isotretinoin, and chromophore gel-assisted phototherapy.^3^^,^^6^^,^^7^ Given that our patient failed many of these medications, a tumor necrosis factor (TNF)-alpha inhibitor, adalimumab, was used off-label because of this disease’s histological granulomatous nature. Blocking TNF-alpha interferes with the maintenance of granulomas and the impairment of macrophage recruitment.^8^ Multiple studies have demonstrated infliximab being an effective treatment for sarcoidosis, which has a similar histology to GR.^8^, ^9^, ^10^ In addition, a retrospective study has demonstrated TNF-alpha inhibitor efficacy in treating granulomatous skin eruptions, such as orofacial granulomatosis, sarcoidosis, and pyoderma gangrenosum.^11^ Although limited improvement was seen in granuloma annulare in this study, case reports have recorded improvement, supporting TNF-alpha therapy as an off-label alternative in other granulomatous cutaneous diseases.^11^^,^^12^

In our case, the effectiveness of this medication was evident at follow up, given the reduction of edema, tenderness, and new cyst formation, indicating a decrease in inflammation overall. Thus, our study highlights TNF-alpha inhibitors as a potential treatment alternative to consider when treating recalcitrant cases of granulomatous rosacea.

## Conflicts of interest

None disclosed.
